# Genetic Homology between Bacteria Isolated from Pulmonary Abscesses or Pyothorax and Bacteria from the Oral Cavity

**DOI:** 10.1128/spectrum.00974-21

**Published:** 2022-02-16

**Authors:** Rinko Katsuda, Junya Inubushi, Haruko Tobata, Toru Eguchi, Kunihiko Terada, Ryogo Kagami, Tetsuji Kawamura, Yoshihiro Kajiwara, Yasuharu Nakahara

**Affiliations:** a Department of Pulmonary Medicine, National Hospital Organization Himeji Medical Center, Himeji City, Hyogo, Japan; b Sunstar Shizuoka Innovation Center, Shizuoka, Japan; c Respiratory Medicine, Terada Clinic, Respiratory Medicine and General Practice, Himeji City, Hyogo, Japan; d Himeji Dental Association Oral Health Center, Himeji City, Hyogo, Japan; University of Illinois at Urbana Champaign

**Keywords:** pulmonary abscess, pyothorax, thoracic infection, bacteria, oral cavity

## Abstract

Pulmonary abscesses and pyothorax are bacterial infections believed to be caused primarily by oral microbes. However, past reports addressing such infections have not provided genetic evidence and lack accuracy, as they used samples that had passed through the oral cavity. The aim of this study was to determine whether genetically identical bacterial strains exist in both the oral microbiota and pus specimens that were obtained percutaneously from pulmonary abscesses and pyothorax, without oral contamination. First, bacteria isolated from pus were identified by 16S rRNA gene sequencing. It was then determined by quantitative PCR using bacterial-species-specific primers that DNA extracted from paired patient oral swab sample suspensions contained the same species. This demonstrated sufficient levels of bacterial DNA of the targeted species to use for further analysis in 8 of 31 strains. Therefore, the whole-genome sequences of these eight strains were subsequently determined and compared against an open database of the same species. Five strain-specific primers were synthesized for each of the eight strains. DNA extracted from the paired oral swab sample suspensions of the corresponding patients was PCR amplified using five strain-specific primers. The results provided strong evidence that certain pus-derived bacterial strains were of oral origin. Furthermore, this two-step identification process provides a novel method that will contribute to the study of certain pathogens of the microbiota.

**IMPORTANCE** We present direct genetic evidence that some of the bacteria in pulmonary abscesses and pyothorax are derived from the oral flora. This is the first report describing the presence of genetically homologous strains both in pus from pulmonary abscesses and pyothorax and in swab samples from the mouth. We developed a new method incorporating quantitative PCR and next-generation sequencing and successfully prevented contamination of pus specimens with oral bacteria by percutaneous sample collection. The new genetic method would be useful for enabling investigations on other miscellaneous flora; for example, detection of pathogens from the intestinal flora at the strain level.

## INTRODUCTION

Although anaerobic bacteria, such as the genus Streptococcus, the species Staphylococcus aureus, and the genus Fusobacterium, have been detected as causative bacteria of lung abscess and pyothorax (1–4), the route of infection has not been fully elucidated. As these pathogenic bacteria are also indigenous oral microorganisms, they are thought to be derived from the oral cavity; however, genetic evidence for an oral origin has not been published. This lack of evidentiary support is because there has been no effective procedure for confirming that bacteria collected from these lesions are also present in the oral microbiota, which contains more than 700 bacterial species (5). Furthermore, bacteria in pulmonary abscesses are commonly collected via the respiratory tract using a bronchoscope, which makes it difficult to avoid contamination of the samples with oral bacteria (6). In addition, anaerobic bacteria may be affected by oxygen during bronchoscopic collection, resulting in a decrease in the recovery yield.

Many papers have discussed causative bacteria based on identification results from culture or a PCR-based method with specific primers; however, such identification does not provide a sufficient basis for causality. Demonstrating genetic homology is essential for clarifying causative bacteria. Owing to the recent advent and advancement of next-generation sequencing (NGS), which has made it possible to easily perform whole-genome sequencing for identification, we developed a novel method to confirm the presence of specific strains in the oral flora with strain-specific primers for PCR. The aim of this study was to determine whether genetically identical bacterial strains existed both in the oral microbiota and in pus specimens that were obtained percutaneously from pulmonary abscesses and pyothorax, without oral contamination.

## RESULTS

### Patient demographic information.

**FIG 1:**
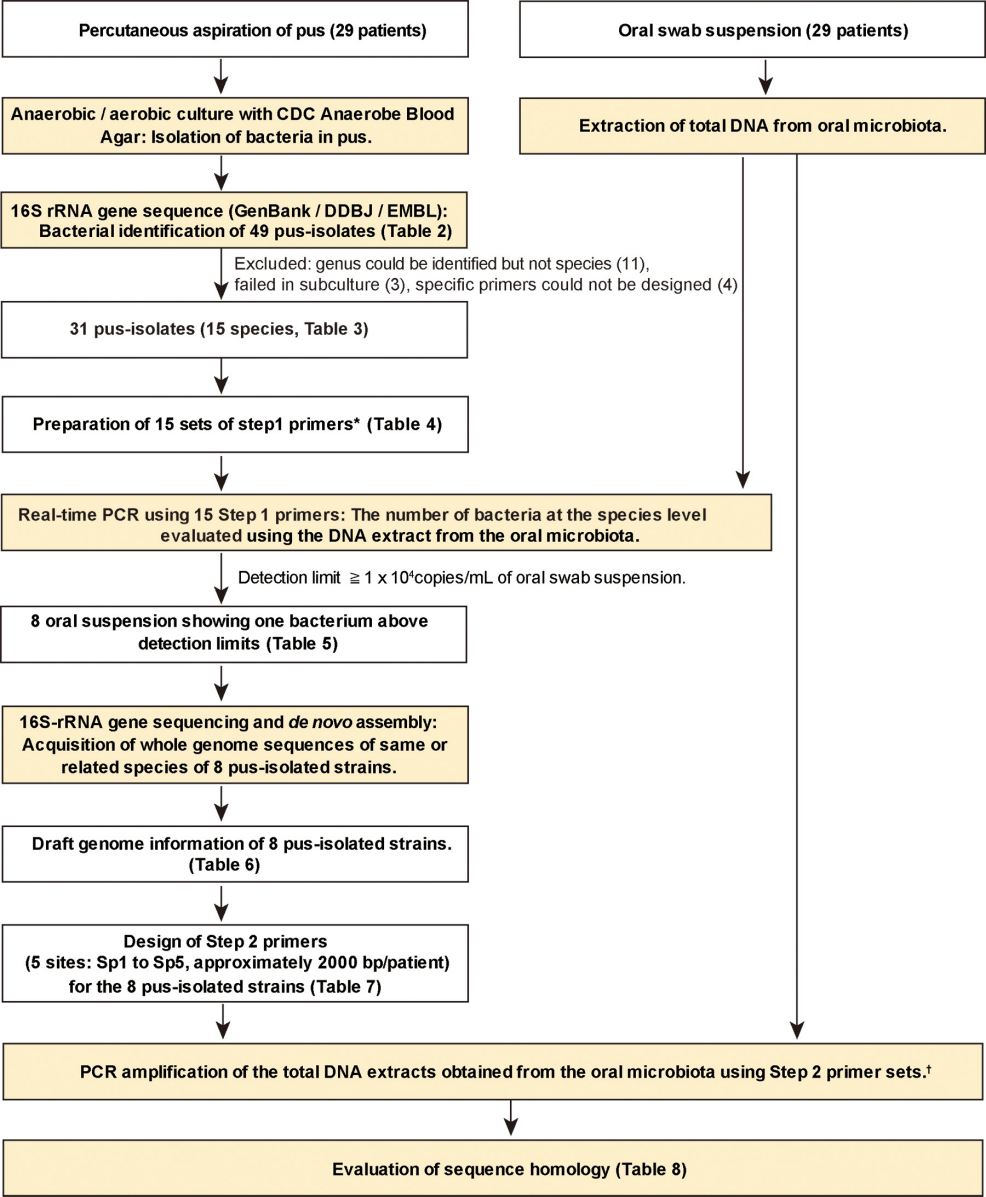
Schematic of the workflow used in the genetic homology analyses of the study. *, Step 1 primers: based on the KEGG database information for the species isolated from pus, we selected or designed step 1 primer sets corresponding to each species isolated from pus. †, Step 2 primers: we designed five strain-specific primers for each strain based on the draft genomes of the strains isolated from pus and the genome information of related species listed in the KEGG database.

Twenty-nine patients (19 with pulmonary abscess and 10 with pyothorax) were enrolled in the study between August 2013 and March 2015. A pus sample and an oral swab sample suspension were collected from each enrolled patient. The demographic information of the patients is shown in Table 1, and the workflow of the study is shown in Fig. 1. The patient cohort consisted of 24 males (15 with pulmonary abscesses and 9 with pyothorax) and 5 females (4 with pulmonary abscesses and 1 with pyothorax). The mean age was 64.6 ± 13.8 years old. The main lifestyle factors included a history of smoking (76%) and high-risk alcohol consumption (34%). The main complications were diabetes mellitus (31%), chronic liver disease (17%), and malignancy (21%). The mean number of remaining teeth was 18.9 ± 9.5, the mean oral hygiene index (OHI) was 2.2 ± 2.4, the mean ratio of periodontal pockets ≥4 mm in depth was 18.6% ± 21.3%, and 21 patients had bleeding from the gingiva.

**TABLE 1:** Demographic and clinical information of the patient cohort

Parameter	Value [no. (%), mean ± SD, or as indicated]
Patient demographics and clinical findings	
Total no. enrolled	29
No. with pulmonary abscess (no. male/female)	19 (15/4)
No. with pyothorax (male/female)	10 (9/1)
Age (yr)	64.6 ± 13.8
Smokers	22 (76)
High-risk alcohol consumption^*a*^	10 (34)
Diabetes mellitus	9 (31)
Chronic liver disease	5 (17)
Malignancy	6 (21)
Oral examination (no. of patients)	
No. of remaining teeth (*n* = 28)^*b*^	18.9 ± 9.5
No. of DMF teeth (*n* = 28)	23.0 ± 7.5
Oral hygiene index (*n* = 28)	2.2 ± 2.4
Ratio of periodontal pockets ≥4 mm deep (%) (*n* = 26)^*c*^	18.6 ± 21.3
Bleeding from the gingiva (yes/no) (*n* = 26)^*c*^	21/5

a High-risk alcohol consumption is 40 g/day in males and 20 g/day in females based on WHO criteria.

b One patient refused a dental evaluation.

c Two of the 28 patients were excluded due to edentulous jaw.

### Identification of strains isolated from pus.

A total of 49 bacterial strains were isolated from the pus samples of 29 patients. Bacterial isolates were identified by 16S rRNA gene sequencing (Table 2).

**TABLE 2:** Identification of 49 bacterial isolates from pus by 16S rRNA gene sequencing

Sample type, patient	Sex^*a*^	Age (yr)	Isolate(s) obtained^*b*^
Pulmonary abscess			
1	F	80	Staphylococcus epidermidis, Staphylococcus pettenkoferi, Slackia sp.^*c*^
2	M	51	Staphylococcus lugdunensis, Propionibacterium acnes
3	M	68	Enterococcus faecalis, Enterobacter aerogenes^*d*^
4	M	84	Fusobacterium nucleatum subsp. *vincentii*^*e*^
5	M	62	Kocuria palustris,^*e*^ Micrococcus luteus or Micrococcus yunnanensis,^*c*^ Parvimonas micra^*e*^
6	M	75	Klebsiella pneumonia
7	M	66	S. epidermidis, Staphylococcus warneri, P. acnes
8	M	58	Streptococcus intermedius
9	F	66	Pseudomonas aeruginosa
10	M	63	Staphylococcus hominis, Fusobacterium sp.^*c*^
11	M	67	Nocardia arthritidis ^*d*^
12	M	35	S. epidermidis
13	M	67	S. intermedius, Staphylococcus caprae or Staphylococcus capitis^*c*^
14	M	81	S. intermedius, Staphylococcus sp.,^*c*^ Eikenella sp.^*c*^
15	M	59	S. caprae or S. capitis^*c*^
16	M	70	S. intermedius, Parvimonas micra^*e*^
17	M	32	Acinetobacter baumannii, S. intermedius
18	F	51	Fusobacterium nucleatum
19	F	68	Parvimonas micra, Actinomyces israelii, Eikenella sp.^*c*^
Pyothorax			
20	M	60	P. aeruginosa
21	M	54	S. warneri
22	M	77	Streptococcus sp.^*c*^
23	M	61	S. intermedius, Mycobacterium sp.^*c*^
24	M	42	S. intermedius
25	M	84	S. warneri, Streptococcus sp.,^*c*^ Gemella morbillorum^*d*^
26	M	89	S. intermedius
27	M	72	S. intermedius
28	M	67	Streptococcus constellatus subsp. *pharynges*
29	F	63	S. warneri, S. constellatus subsp. *constellatus*

a F, female; M, male.

b Thirty-five isolates derived from pulmonary abscess in 19 patients and 14 from pyothorax in 10 patients. Of the 49 bacterial isolates, we designed step 1 primer sets for 31 isolates, including 15 species. The remaining isolates were excluded from primer design for reasons specified in footnotes *c* to *e*.

c Isolate could only be identified at the genus level.

d Isolate lacked genome information of related species in the KEGG database or isolate-specific primers could not be designed.

e Isolate failed in subculture.

### Evaluation of genetic homology.

A schematic of the workflow used to evaluate genetic homology in the current study is shown in Fig. 1. To determine whether the same bacterial strains were present in both the pus samples and oral swab sample suspensions of individual patients, we performed a two-step process that included the use of both “step 1 primer sets” and “step 2 primer sets.” Among the 49 bacterial isolates (Table 2), we designed step 1 primer sets for 31 bacterial isolates that included 15 bacterial species (Table 3), and we excluded 11 isolates that could only be identified to the genus level, 3 isolates that failed in subculture, and 4 isolates that lacked genome information of related species in the KEGG database or whose specific primers could not be designed (Table 4). The homology rates of the selected 31 bacterial isolates were 99.2 to 100%. Evaluation of the samples by quantitative PCR (qPCR) using the step 1 primer sets showed that eight oral swab sample suspensions included more than 1 × 10^4^ copies/swab sample of the same species as were in the patient-matched pus isolates (Table 5). These bacterial species included Streptococcus intermedius (2 patients), Fusobacterium nucleatum, and Parvimonas micra from pulmonary abscesses and S. intermedius (2 patients), Streptococcus constellatus subsp. *pharyngis*, and Streptococcus constellatus subsp. *constellatus* from pyothorax. This indicated that identical strains may be present in both the pulmonary abscesses or pyothorax pus and the oral cavity microbiota.

**TABLE 3:** The 15 species used for preparing step 1 primer sets and patients in whom the species were detected

Isolate	Patient^*a*^
Staphylococcus epidermidis	1, 7, 12
Staphylococcus pettenkoferi	1
Staphylococcus lugdunensis	2
Propionibacterium acnes	2, 7
Enterococcus faecalis	3
Klebsiella pneumonia	6
Staphylococcus warneri	7, 21, 25, 29
Streptococcus intermedius	8, 13, 14, 16, 17, 23, 24, 26, 27
Pseudomonas aeruginosa	9, 20
Staphylococcus hominis	10
Acinetobacter baumannii	17
Fusobacterium nucleatum	18
Parvimonas micra	19
Actinomyces israelii	19
Streptococcus constellatus	28, 29

a Underlining shows a pyothorax patient.

**TABLE 4:** Sequences of the 15 species-specific step 1 primer sets^*a*^

Isolate	Target gene	Primer sequence (5′→3′) or kit used	
		Forward	Reverse
S. epidermidis	*gmk*	ATGCGTTGTCATATTTTTAGCGCCTCCA	CAACAAGACGTTCTYTCAAGTCATCT
S. pettenkoferi	*rpoB*	AGAATGCACTTAAGAATCTCGAC	AGCGTGTAATAAGCGCTCTTCG
S. lugdunensis	*rpoB*	TTAGTACGCGTATATATTGTGCAG	AAATAAGGCATGTCCTCTTCT
P. acnes	*grpE*	GCCGAATACGTCAACTACAAGAGG	CGAAACTGGTCAGGCCGTGATTAT
E. faecalis	*grpE*	E. faecalis detection kit, catalog no. RF-0003; TechnoSuruga Laboratory	
K. pneumonia	*fimD*	CAGCGATTCTCGTTGGATGAGGT	CCAGGCATTTATGGGTAACCATGCG
S. warneri	*sodA*	TGTAGCTAACTTAGATAGTGTTCCTTCT	CCGCCACCGTTATTTCTT
S. intermedius	*groEL*	CATCAGCYTCCAGTTCAAGAGCAG	GTGGTGTGGCTGTCATCAAAGTA
P. aeruginosa	*gyrB*	CCTGACCATCCGTCGCCACAAC	CGCAGCAGGATGCCGACGCC
S. hominis	*rpoB*	GTTACCACGGAAACGACATACC	CGAAACACCATATCGTAAAGTAAAGTAGATATT
A. baumannii	*rpoB*	GAAGAAATTCTAGCGCTTGCAGGT	CGTACGTGAAATGTCAGCCAAT
F. nucleatum	16S rRNA gene	CTTAGGAATGAGACAGAGATG	TGATGGTAACATACGAAAGG
P. micra	16S rRNA gene	TCGGGAATGGAAATGAAATGAAAG	TGGTCTCATGCGGTATTAATCGTC
A. israelii	16S rRNA gene	ATGCAAGTCGAACGGGTCTG	AAGTGAATCTTTCCCAGCCACA
S. constellatus	*groEL*	TCCAAAGCACGAAGAACGATATTA	TTGTGGAAGGTTCAGGAGC

a Based on the reference information for each of the strains isolated from pus, we selected or designed step 1 primers corresponding to each pus-isolated species. The DNA extracted from the oral swab sample suspension of each patient was examined using step 1 primers and qPCR.

**TABLE 5:** Results of qPCR analysis using the 15 step 1 primer sets corresponding to the pus isolates on the oral swab sample suspension derived from each patient

Source of pus isolate, patient	Sex^*a*^	Age (yr)	Step 1 primer set target species^*b*^	qPCR result (copies/swab sample)^*c*^
Pulmonary abscess				
16	M	70	S. intermedius	1.21 × 1E+5
17	M	32	S. intermedius	1.00 × 1E+5
18	F	51	F. nucleatum ^*d*^	1.73 × 1E+7
19	F	68	P. micra ^*d*^	1.22 × 1E+6
Pyothorax				
26	M	89	S. intermedius	2.36 × 1E+4
27	M	72	S. intermedius	9.25 × 1E+5
28	M	67	S. constellatus	9.00 × 1E+6
29	F	63	S. constellatus	2.10 × 1E+7

a M, male; F, female.

b See Table 4 for the primer sets.

c The detection limit was ≥1 × 10^4^ copies/swab sample.

d Anaerobic bacterium.

**FIG 2:**
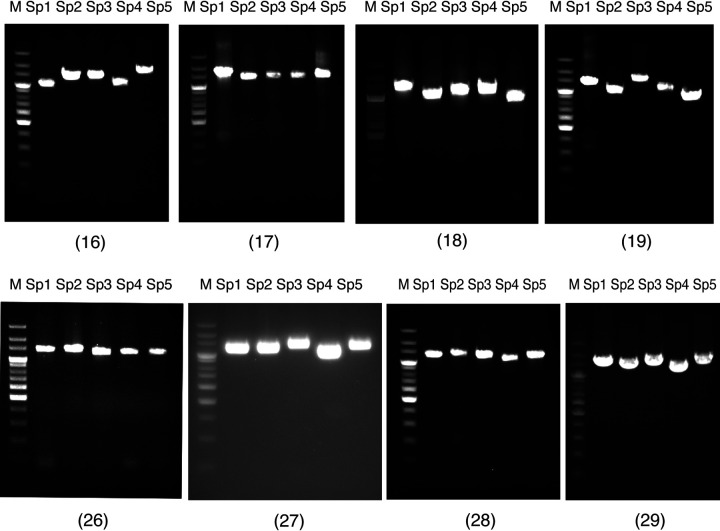
Representative agarose gel electrophoresis results showing PCR amplicons generated using the total DNA extracts obtained from the oral microbiota of patients 16 to 19 and 26 to 29 and the five step 2 primer sets Sp1 to Sp5. Lanes M show a 200-bp ladder. Amplified fragments of the expected size (see Table 7) were obtained for total DNA extracts in all samples (16 to 19 and 26 to 29).

Next, we performed genome sequencing and *de novo* assembly of the eight strains isolated from pus. The genome sizes determined by *de novo* assembly of the draft genomes for the strains isolated from pus and their DDBJ accession numbers are shown in Table 6. We then designed five step 2 primer sets (strain-specific primers Sp1 to Sp5) for use in amplifying strain-specific sequences (approximately 2,000 bp/site). The step 2 primer sets were designed for each of the strains based on the draft genomes of the strains isolated from pus and genomic information. Total DNA extracted from the oral cavity swab sample suspensions of each of the eight patients was PCR amplified using the five step 2 primer sets for each strain (Table 7). Representative agarose gel electrophoresis results of the PCR amplicons are shown in Fig. 2. Amplified fragments of expected sizes were obtained for the total DNA from eight of the oral swab sample suspensions. The PCR amplicons from the oral microbiota samples were sequenced, and the results compared to the sequences of the pus isolates, as well as to those of the reference sequences from the GenBank, DDBJ, and EMBL databases. All PCR amplicon sequences of the oral microbiota samples completely matched (100% identity) the sequences from the strains isolated from pus. This confirmed that the sequences were identical between the strains isolated from pus and the oral amplicons, and there were no single-nucleotide polymorphism (SNPs), indels, or other variations identified. In contrast, there were no 100% matches for the sequences available in the open databases, for which homology ranged from 67% to 99.3% (Table 8).

**TABLE 6:** Results of whole-genome sequencing and *de novo* assembly^*a*^

Patient^*b*^	Pus isolate	Used reads/obtained reads^*c*^	Genome size (bp)	DDBJ accession no.
16	S. intermedius	4,875,953/5,931,508	1,845,135	BHYS01000001–BHYS01000008
17	S. intermedius	3,749,825/5,437,372	1,813,578	BHYV01000001–BHYV01000007
18	F. nucleatum	3,807,625/4,947,250	2,019,333	BHYR01000001–BHYR010000041
19	P. micra	4,611,319/5,891,488	1,634,483	BHYQ01000001–BHYQ01000014
26	S. intermedius	4,015,112/5,322,984	1,859,692	BHYT01000001–BHYT01000026
27	S. intermedius	15,241,529/19,558,444	1,946,261	BHYP01000001–BHYP01000006
28	S. constellatus subsp. *pharyngis*	3,243,579/4,521,796	1,914,344	BHYU01000001–BHYU01000013
29	S. constellatus subsp. *constellatus*	17,283,453/22,962,102	1,800,973	BHYO01000001–BHYO01000016

a Genome sizes and accession numbers of contiguous sequences of eight strains isolated from pus in pulmonary abscess/pyothorax, obtained by *de novo* assembly.

b Patients 16 to 19 had pulmonary abscesses; patients 26 to 29 had pyothorax.

c Reads used in *de novo* assembly and their contribution to contiguous sequence creation.

**TABLE 7:** Sequences of strain-specific step 2 primer sets^*a*^

Patient	Site	Step 2 primer sequence		Size of PCR amplicon (bp)
		Forward	Reverse	
16	Sp1	GATGAAACACCTATTTTGCTCTACTGT	AGCTCACCTGGCTCAATATTTA	2,110
	Sp2	TGGAACGGATATGAATGGGACTCT	GATACCGTGACGGATAGCTCCTGATT	2,414
	Sp3	AATCTCCCTATAAGAGTCGCCGATTG	TTTACCGTCCGCATAAGTTCCAGCAT	2,351
	Sp4	AGTTAGTGCTGGCGGACAAG	ATGTTTTAGGTGCCACTACCCG	2,066
	Sp5	AACAAAGAGGCTCTGGAAAGGC	GGAAGACCTGAACCAATCCT	2,644
17	Sp1	AGAAGGCGTGAGGTTAAGAGTC	CCAGTTACTTGTTTAGCCCGTTTGTC	2,551
	Sp2	TTAAGCCCTAGAACGCCGGAAGTA	GCTATACAAATTCCCAGAAAACCCA	2,374
	Sp3	AAGTCGTTGGACAGTCTATCATGAAG	GGCATAGTACTTTTAGTAATGAATCT	2,435
	Sp4	TTTTGGTTTCATAATTCCTCCAGTA	ACCACACCAATTACTCTAGGGG	2,469
	Sp5	GATTGAGCAATGGCACGTTTTACAAG	TGAAGAATTAGCACTGCGTAGCCG	2,420
18	Sp1	AACATGGCTGGGTGTAAAGCTA	GGCAAGATTTTCTGAAGCTACA	2,333
	Sp2	ACTAGCTGGGAAAGGTGCTCT	CAATGTTTGGAATGTCAATAGGAGC	2,031
	Sp3	TTTTCTAAGAGCCACTTCACTAC	TAATATTTCCAGCATTGACACCTTC	2,150
	Sp4	AGATATGTCTACGCCTTCTTCCCC	TTCCCTGCTTTTAATTCTTCTGAATA	2,210
	Sp5	GGCACAAGGTTTAAAAGCTCCGAC	ATACTTTTCCTTTGTTTGTTGCTTTTCCATTTGGT	1,859
19	Sp1	GTTAAAACTTCAAGCTTTCCGTCA	ACAGGGGACAAGGCTAAAA	2,375
	Sp2	TGTAAGTTGAATTTTGACAAGGGT	CTAAAATTCCAAGCCCTCTAATCTC	2,030
	Sp3	GGTGGACCTAATGGAGATGTA	GTCTTGACTTGATAGGTCCCCTTG	2,579
	Sp4	GGAGTTTTGAATTTCGAAGAAATCCC	TTCCTGAAAGGTTATTTTCTTTACAT	2,202
	Sp5	CGGAAGCTTGGAAAAATTATTGCTCA	CAAACATAACAGCTTTTTCTTTGTTC	1,929
26	Sp1	TCCAAATCTGACTGGGCCACCAT	CCACCGACGGATAGTGAGGTAGGTTA	2,416
	Sp2	GCGGCATGAGTGAAGAAAACACGA	CTCCAAGATGCGGTCAATGTCAAAGC	2,402
	Sp3	CGAAAATGTCCCACGGATGTTTGC	CTTACCGTTGAGCTGCTTAGCAGA	2,266
	Sp4	TGGGGATTAAACCAGCCACGAAAG	CATCAGGACCAATAAAGGTCCAAGCA	2,298
	Sp5	CACGTTACCAACGACAGACGGC	TTCCGCCTTGTAAGCCGCAAA	2,300
27	Sp1	GGAGGATATGCCTGAACTTGCCTT	ACGAATCCGAGCAATGTAACCC	2,279
	Sp2	CAGTCTGTGCTGGAAAAGCTGGAT	TTGGTCGATTTGCCTTGACCCAT	2,252
	Sp3	AGGACAACTACATGCAACAGTTGCT	GCAATGCAAAGAACAGGGCAGGAT	2,486
	Sp4	TATTTGCTGTCGCAGGCTGATGGT	TTACCAGCACTACAAGCCACAAGAG	2,090
	Sp5	GGCTAAAGCAGCTAGCGAAGCTAT	GAAATTCCTCCACACCAGATTCGC	2,295
28	Sp1	TTTTCCTTTGGTCAATTTAGAAACCC	TATTCAGAGACTTTTCAGAGGTA	2,270
	Sp2	AGCTGATAGAGGTCATAGTGCTCTCG	TGATTGGGCAGATTTCACGAAGCTC	2,352
	Sp3	AGCGTAAATAAACTTAGACTTTCTT	CGACTCCAGCTAATGCTCCTAT	2,261
	Sp4	AACAGTCTGAAAGATAGGATTAACAC	ATTCTCACCAGAAGCCAAAGCAG	2,095
	Sp5	ATCTCTTGCTCGTCGACCCCAAAT	GGCTTATAAAGCACGTTTTCGAGTAA	2,220
29	Sp1	TATTCGCCGCGATCCCATTTG	CACTCCGACTAGCACAACCGCTGAA	2,513
	Sp2	ACCCGCTTTGAACGGAGAT	GGCTAAAATAACTGTTCCTATGC	2,492
	Sp3	GCTTGACTGAAGAACAGGCAATCC	CGAAATTTTAGTTTGTTAACCGGGC	2,631
	Sp4	ACCACATCTCAAACTTATCCCTCTT	CGCCCATCCGCAATTCCG	2,299
	Sp5	AAACACGCCAGATTATCGGCGCA	GTCAGTTTAGATTTCTGCTGGGCAT	2,632

a We designed 5 strain-specific primers (5 sites, Sp1 to Sp5, approximately 2,000 bp/patient) based on the draft genomes of the strains isolated from pus (14) and the genomic information of related species listed in the KEGG database.

**TABLE 8:** Homology of PCR amplicons from oral swab samples to the best-matching candidate strains in an open database and strains isolated from pus

Source of pus isolate, patient	Pus isolate	Best-matching candidate strain in an open database to amplicon from oral swab sample, % homology^*a*^					% homology of amplicons of Sp1–Sp5 to strain isolated from pus^*b*^
		Sp1	Sp2	Sp3	Sp4	Sp5	
Pulmonary abscess							
16	Streptococcus intermedius	S. intermedius C270	S. intermedius C270	S. intermedius C270	Streptococcus anginosus J4206	S. intermedius JTH08	100
		99.40	99.30	96.60	94.80	80.40	
17	S. intermedius	S. intermedius TYG1620	S. intermedius JTH08	S. intermedius KCOM1545	S. intermedius C270	S. intermedius C270	100
		99.60	99.10	97.00	95.20	95.00	
18	Fusobacterium nucleatum	F. nucleatum subsp. *polymorphum* NCTC10562	F. nucleatum subsp. *animalis* 7_1	F. nucleatum subsp. *animalis* 7_1	F. nucleatum subsp. *polymorphum* ChDC F306	F. nucleatum subsp. *nucleatum* ChDC F316 KCOM1322	100
		94.90	93.40	90.40	93.20	89.30	
19	Parvimonas micra	P. micra KCOM1535 ChDC B708	P. micra KCOM1535 ChDC B708	P. micra KCOM1535 ChDC B708	P. micra KCOM1535 ChDC B708	P. micra KCOM1535 ChDC B708	100
		98.60	95.60	95.20	91.00	84.90	
Pyothorax							
26	S. intermedius	S. intermedius KCOM1545	S. intermedius KCOM1545	S. intermedius JTH08	S. intermedius JTH08	S. intermedius C270	100
		98.10	93.60	93.60	89.00	94.60	
27	S. intermedius	S. intermedius B196	S. intermedius B196	S. intermedius B196	S. intermedius B196	S. intermedius JTH08	100
		99.30	98.50	97.70	92.00	97.20	
28	Streptococcus constellatus subsp. *pharyngis*	S. anginosus C238	S. constellatus subsp. *pharyngis* C1050	S. constellatus subsp. *pharyngis* C1050	S. anginosus J4206	S. anginosus subsp. *whileyi* MAS624	100
		99.90	96.60	95.70	95.70	95.50	
29	S. constellatus subsp. *constellatus*	S. constellatus subsp. *pharyngis* C1050	S. constellatus subsp. *pharyngis* C1050	S. constellatus subsp. *pharyngis* C1050	S. intermedius B196	Streptococcus gallolyticus subsp. *gallolyticus* ATCC BAA-2069	100
		98.30	97.10	81.20	96.50	93.00	

a GenBank, DDBJ, and EMBL were the databases used.

b The sequences of the five amplicons were all 100% matched with the five specific sequences from the strains isolated from pus.

## DISCUSSION

In the current study, we demonstrated for the first time that genetically identical strains were present in both pulmonary abscess and pyothorax pus samples and swab samples of the oral microbiota. Furthermore, we were able to validate a new NGS-based approach for this type of study. We isolated 49 bacterial strains from the lesions of 29 patients by 16S rRNA gene sequencing, but we failed to determine the species for some isolates. Nevertheless, the 49 isolated bacterial strains included representatives of the reported causative microorganisms of pulmonary abscess or pyothorax, such as Staphylococcus, Streptococcus, Parvimonas, Pseudomonas, and Fusobacterium (1–4). Among these isolates, four strains from pulmonary abscesses and four strains from pyothorax were subjected to our novel genetic homology analysis. PCR amplification of total DNA extracted from oral swab sample suspensions showed that the oral microbes shared 100% sequence homology with their patient-matched pus sample counterparts.

Anaerobes and microaerophilic streptococci have been suggested to be the causative organisms in most cases of pulmonary abscess or pyothorax, as the disease can be reproduced using anaerobes recovered from the gingival crevice and since multiple studies of transtracheal and transthoracic aspirates consistently implicate these bacteria (1, 2). However, no report to date has shown genetic homology between clinical isolates from these types of lesions and the oral microbiota, which has largely been due to the lack of appropriate techniques. The “gold standard” method for determining genetic homology is pulsed-field gel electrophoresis, but this does not allow one-to-many DNA pattern comparisons between clinical isolates and the entire microbiota.

In this study, we developed a novel method for evaluating genetic homology. Whole-genome sequences of strains isolated from pus were created using an NGS method and then compared with those of the same or related species available in publicly accessible databases in order to develop strain-specific primer sets. To increase precision, five sets of strain-specific primers were synthesized for each strain isolated from pus. Total DNA extracted from oral swab sample suspensions was then amplified using these strain-specific primers, with all samples leading to the generation of PCR amplicons. This suggested that there were identical sequences in both the strains isolated from pus and the oral microbiota. To confirm this, each PCR amplicon was sequenced and the results used to match sequences from the oral microbiota to those of the strains isolated from pus. Our findings provide the first evidence of genetically identical bacteria being present in both pulmonary lesions and the oral cavity.

Many bacterial species have been linked to pulmonary abscesses and pyothorax (1–4). This is especially true for the Streptococcus anginosus group, consisting of S. intermedius, S. constellatus, and S. anginosus, which are common indigenous microbiota of the oral cavity and have all been shown to cause deep-seated organ abscesses (7–13). The S. anginosus group of microorganisms accounted for 35.5% (11/31 strains) of the bacterial isolates identified in the current study. Staphylococcus species were the next most frequently isolated bacterial strain (25.8%, 8/31 strains); however, we were unable to detect these bacterial isolates in the oral microbiota (Table 2). Thus, we suspect that these Staphylococcus isolates probably originated from the nasal cavity through the pharynx.

Bacteriological studies of pulmonary abscesses have always experienced significant challenges in sampling specimens without contamination from the oral cavity and oxygen exposure. Transtracheal aspiration and protected bronchial brushing are the most common procedures used to collect uncontaminated specimens (14). In the current study, we sampled pus directly from the pulmonary abscess using percutaneous fine-needle aspiration (FNA) under X-ray fluoroscopic guidance, thereby completely avoiding the potential for contamination from the oral cavity. In addition, because our procedure was completed within one breath of the patient, it reduced the risk of air embolism and pneumothorax to the patient compared to the risk for computed tomography (CT) guidance and also minimized the effects of oxygen on the anaerobic bacteria.

We collected demographic information of the patients and performed oral examinations and found that the oral hygiene of the 29 patients in our study did not deteriorate: the mean ratio of periodontal pockets ≥4 mm in depth was 18.6% ± 21.3% and the mean OHI was 2.2. However, the mean number of decayed, missing, and filled (DMF) teeth (23 ± 7.5) was higher than that of the general Japanese population (17.1 for the age range of 55 to 64 years and 19.2 for the age range of 65 to 74 years) and the mean number of residual teeth (18.9 ± 9.5) was lower than the Japanese average (23.9 for the age range of 60 to 64 years and 21.6 for the age range of 65 to 69 years) (15). This indicates the relatively poor oral hygiene conditions of the patients and the possibility of complicating oral infectious sites, such as apical abscesses. The causative bacteria of purulent lung infections, such as F. nucleatum, P. micra, and S. anginosus, have been detected more often in apical abscesses than in saliva (16–18). In our study, we used oral suspensions to assess the oral microbiota, and we detected the species isolated from pus from a sufficient amount of bacterial DNA with the step 1 primers in only eight patients. If we had used samples from the possible infectious sites in the oral cavity, we might have been able to detect bacteria in a higher number of patients.

The current study has some limitations, in addition to the above-mentioned oral sampling method. The purpose of the study was to determine whether any causative bacteria of pulmonary abscesses or pyothorax were also present in the oral microbiota of the patient. That noted, we did not examine all possible causative bacteria. In other words, we did not consider bacteria that could not be cultured from the pus samples or were not listed in the KEGG database. Further studies would be helpful in creating a more robust data set.

In conclusion, our study provides the first demonstration that, in some cases, strains isolated from pus from a pulmonary abscess and pyothorax are genetic matches for DNA extracted from paired patient oral swab sample suspensions and, thus, are of oral origin. This finding provides strong evidence for the importance of oral care in preventing pulmonary abscesses and pyothorax. In addition, this new approach can be applied to other studies designed to detect pathogenic bacteria at the strain level in promiscuous microbiota associated with different diseases and is a robust tool for evaluating the underlying pathology of various infectious diseases.

## MATERIALS AND METHODS

### Study participants.

This study was conducted after approval by the institutional review board of Himeji Medical Center on 21 June 2013. The enrolled participants included 29 patients with pulmonary abscess (*n* = 19) or pyothorax (*n* = 10) who visited the Himeji Medical Center respiratory medicine department between August 2013 and March 2015. Patients were assigned to the pulmonary abscess group when they presented pulmonary infections with a mass-like consolidation in the thoracic cavity or low-density areas within the lesions upon examination using contrast-enhanced chest CT. Those patients then underwent sampling of their lesions for isolation of bacteria. Patients were diagnosed with pyothorax when they exhibited symptoms of fever and pleural effusions, including bacterial contaminants.

### Sample collection.

For the collection of pus from pulmonary abscesses, the distance between the lesion and the skin was first measured using CT. Pus was collected by FNA using a 22-gauge needle inserted percutaneously under X-ray fluoroscopic guidance at the depth measured in advance on CT. The procedure was performed while the patient held a single breath for approximately 20 s. Pus samples from patients with pyothorax were collected from the thoracic cavity using a fine needle. Oral swab samples were obtained by scraping the whole oral cavity with an oral swab (Butler SG Sponge Brush; Sunstar, Inc., Osaka, Japan) wetted with sterile water.

### Oral examination.

Within 14 days of sampling, the number of residual teeth, the number of teeth with caries experience (decayed, missing, and filled [DMF] teeth), the oral hygiene index (OHI) (19), periodontal pocket depths (20), and bleeding on probing from subgingival pockets were evaluated by dentists.

### Isolation of bacterial strains from pus samples.

Each sample was smeared onto two plates of CDC anaerobe blood agar (Becton, Dickinson Japan, Tokyo, Japan). The plates were then incubated under aerobic or anaerobic conditions, and the resultant bacterial strains were isolated as pure cultures.

### Identification of bacterial strains isolated from pus using 16S rRNA gene sequencing.

Strains isolated from pus were identified by TechnoSuruga Laboratory (Surugaku, Shizuoka City, Shizuoka, Japan). The total length of 16S rRNA (approximately 1,500 bp) was amplified by PCR using the primer pair 9F/1406R, and then a sequence homology search was performed using partial sequences of approximately 500 bp on the 9F side to identify bacterial species. The homology search was performed using Apollo 2.0 DB-BA9.0 software (TechnoSuruga Laboratory) and sequences from the GenBank, DNA Data Bank of Japan (DDBJ), and European Molecular Biology Laboratory (EMBL) databases.

### DNA extraction from oral swab samples.

The tips of the oral swabs were aseptically cut from the swab shaft and mixed with 20 mL of sterile phosphate-buffered saline (PBS). After stirring this solution and removing the swab, it was centrifuged at 4°C and 13,000 rpm for 10 min to obtain pellets containing oral bacteria. The pellets were resuspended in 1 mL sterile PBS, and total DNA was extracted from 100 μL of the oral swab sample suspension using a Mora-extract DNA extraction kit (Kyokuto Pharmaceutical Industrial Co., Ltd.). The extracted DNA was resuspended in 100 μL Tris-EDTA (TE) buffer, and a final DNA solution at 10 ng/μL was prepared in TE buffer.

### qPCR using step 1 primer sets.

As the initial step, we determined at the species level whether the same organisms were present among both the strains isolated from pus and the oral swab sample suspensions. Based on the information in the KEGG database regarding the species isolated from pus (21–24), we selected or designed species-specific step 1 primers. The extracted DNA from the oral swab sample suspensions was evaluated using qPCR and the step 1 primers at TechnoSuruga Laboratory according to the method described by Takahashi et al. (25). The lower limit of detection of the PCR assay was 1 × 10^4^ copies/mL.

### Acquisition of draft genomes of strains isolated from pus (genome sequencing and assembly).

To determine the presence of the strains isolated from pus in the oral cavity at the strain level, we acquired draft genomes of the strains whose species were detected in the oral swab sample suspension using the above-described step 1 primer assay. Genomic DNA was extracted from the strains isolated from pus. After purifying DNA fragments from the agarose gel to remove contaminants, the genomic DNA was fragmented (550-bp insert) using the standard protocol 15041110 rev.C (December 2014) with the Illumina TruSeq nano DNA sample preparation kit (26), and then paired-end sequencing (2 × 300 bp) was performed with the Illumina MiSeq to obtain a base sequence, according to the manufacturer’s instructions. Genome information from the strains isolated from pus was obtained using Velvet (version 1.2.08) software for *de novo* assembly of the fragmented sequence data. This series of data were obtained by Hokkaido System Science Co., Ltd. (Sapporo City, Hokkaido, Japan).

### Design of step 2 primer sets for strains isolated from pus.

We designed step 2 primer sets in accordance with the method described by Bru et al. (27). Based on the draft genomes of the strains isolated from pus and the genomic information from the related species deposited in KEGG, we searched for five sites with strain-specific sequences for each strain isolated from pus (approximately 2,000 bp/site) and designed five step 2 primer sets (Sp1 to Sp5), which included both forward and reverse primers, for amplifying the strain-specific sequences.

### PCR amplification using step 2 primer sets and evaluation of sequence homology.

PCR amplification of total DNA extracted from the oral swab sample suspensions was performed at TechnoSuruga Laboratory using the five step 2 primer sets. The PCR mixture (25 μL) contained 20 ng of DNA, 1.25 units of TaKaRa LA *Taq* DNA polymerase, 3 μL of each primer (2.5 μM), 2.5 μL of 10× LA PCR buffer II (Mg^2+^ free), 4 μL of deoxynucleoside triphosphate (dNTP) mixture (2.5 mM), and 2.5 mM MgCl_2_. The cycling conditions were as follows: initial denaturation at 94°C for 1 min, followed by 35 cycles of 94°C for 30 s, 55°C for 30 s, and 72°C for 3 min, and then a final extension at 72°C for 8 min. The PCR amplicons were evaluated using agarose gel electrophoresis. The sequences of the PCR amplicons were compared to the sequences from the same or related species available in the GenBank, DDBJ, and EMBL databases using a sequence homology search.

### Data availability.

The draft genomes of the eight strains isolated from pus and reported in this study, including S. anginosus group (*n* = 6), P. micra (*n* = 1), and F. nucleatum (*n* = 1), have been deposited in DDBJ under the following accession numbers: S. intermedius (patient 16), BHYS01000001 to BHYS01000008 (https://www.ncbi.nlm.nih.gov/nuccore/BHYS00000000.1); S. intermedius (patient 17), BHYV01000001 to BHYV01000007 (https://www.ncbi.nlm.nih.gov/nuccore/BHYV00000000.1); S. intermedius (patient 26), BHYT01000001 to BHYT010000026 (https://www.ncbi.nlm.nih.gov/nuccore/BHYT00000000.1); S. intermedius (patient 27), BHYP01000001 to BHYP01000006 (https://www.ncbi.nlm.nih.gov/nuccore/BHYP00000000.1); S. constellatus subsp. *pharynges* (patient 28), BHYU01000001 to BHYU010000013 (https://www.ncbi.nlm.nih.gov/nuccore/BHYU00000000.1); S. constellatus subsp. *constellatus* (patient 29), BHYO01000001 to BHYO010000016 (https://www.ncbi.nlm.nih.gov/nuccore/BHYO00000000.1); P. micra (patient 19), BHYQ01000001 to BHYQ010000014 (https://www.ncbi.nlm.nih.gov/nuccore/BHYQ00000000.1); and F. nucleatum (patient 18), BHYR01000001 to BHYR010000041 (https://www.ncbi.nlm.nih.gov/nuccore/BHYR00000000.1).
